# Acceptability Limits for Inter-instrument Variability in Viral Serological Tests on Two Enhanced Chemiluminescence Immunoassay Instruments: Evidence From a Hospital Laboratory-Based Study

**DOI:** 10.7759/cureus.102181

**Published:** 2026-01-23

**Authors:** Sandeep Thirunavukkarasu, Mallikarjun Suligavi, Rakesh B Anand, Prasanthi Sanjeevi, Rupashi Vaid, Shanu Sharma

**Affiliations:** 1 Microbiology and Infectious Diseases, BGS Medical College and Hospital, Bengaluru, IND; 2 Biochemistry, BGS Medical College and Hospital, Bengaluru, IND; 3 General Surgery, BGS Medical College and Hospital, Bengaluru, IND; 4 Biostatistics, BGS Medical College and Hospital, Bengaluru, IND; 5 Microbiology, Ramaiah Medical College and Hospital, Bengaluru, IND; 6 Microbiology, BGS Medical College and Hospital, Bengaluru, IND

**Keywords:** analytical validity, chemiluminescent immunoassay, hepatitis b, hepatitis c, human immunodeficiency virus, inter-instrument comparison

## Abstract

Background

Reliable viral serological testing is essential for accurate diagnosis and public health management of HIV, hepatitis B, and hepatitis C. Chemiluminescent immunoassay platforms, particularly enhanced chemiluminescence (eCLIA) systems, are widely used; however, inter-instrument variability remains a challenge in laboratories operating multiple analyzers. Defining acceptable limits of variation is crucial for ensuring diagnostic accuracy, laboratory quality assurance, and clinical decision-making.

Objectives

To evaluate the correlation and agreement between two eCLIA platforms (Vitros ECi and Vitros 3600) in detecting HIV, HBsAg, and HCV, and to establish data-driven acceptability limits for inter-instrument variability.

Methods

An analytical cross‑sectional study was conducted in a diagnostic laboratory of a medical college in Bengaluru, India (January 2019-December 2024). Each day, one patient serum sample was selected at random and tested simultaneously on both eCLIA analyzers. Hemolyzed, lipemic, or insufficient samples were excluded. This random sampling approach, with one sample per day, was considered appropriate for inter‑instrument comparison, as most accreditation bodies recommend testing 1-2 samples only every three months. Results were expressed as S/CO values. Descriptive statistics, Pearson correlation, and Bland-Altman analyses were performed. Jarque-Bera testing assessed normality. Bootstrapping (1000 iterations) was conducted to evaluate the stability of out-of-limit findings and propose acceptable error thresholds.

Results

Of 830 samples processed, the final dataset (after excluding incomplete or zero values) showed high variability across parameters (CV > 50%; HCV > 100%). Pearson correlation demonstrated strong linear relationships for HCV (r = 0.85, R² = 0.72) and HBsAg (r = 0.85, R² = 0.73), but only moderate correlation for HIV (r = 0.64, R² = 0.41). Jarque-Bera statistics (p<0.0001) indicated non-normal distribution, supporting the use of Bland-Altman analysis. Bland-Altman plots revealed approximately 95% agreement for all three markers, with similar proportions of out-of-limit values despite differing correlation strengths. Bootstrapping analysis showed ≤5% error across simulated datasets, suggesting a 5% inter-instrument variability as a reasonable acceptability limit.

Conclusion

Correlation alone is insufficient to assess agreement between CLIA instruments, particularly for non-normally distributed diagnostic data. Bland-Altman analysis combined with bootstrapping provides a more robust approach for evaluating inter-instrument variability. Based on empirical error estimates, an acceptability threshold of ≤5% variability is proposed for laboratories using dual eCLIA platforms for viral serology. These findings support evidence-based quality assurance and instrument interchangeability in clinical diagnostics.

## Introduction

Accurate and early detection of infectious diseases remains a cornerstone of effective public health strategies and clinical management. Human immunodeficiency virus (HIV), hepatitis B virus (HBV), and hepatitis C virus (HCV) continue to pose significant global health challenges. Timely and reliable diagnosis is crucial for individual patient care and plays a pivotal role in epidemiological control and prevention strategies

Chemiluminescent immunoassay (CLIA) technology has been widely used for detecting viral infections due to its enhanced sensitivity, specificity, rapid turnaround time, and automation capabilities. Diagnostic laboratories use this method to perform serological tests for detecting antibodies to HIV and HCV, as well as Hepatitis B surface antigen (HBsAg). With ongoing advancements in laboratory instrumentation, multiple CLIA-based platforms are now available, including the enhanced chemiluminescence immunoassay (eCLIA) systems integrated into the Vitros analyzers by QuidelOrtho, which are commonly used in clinical settings [1].

Many laboratories maintain two or more instruments for serological tests, as accreditation agencies require contingency plans for equipment breakdowns. Each instrument has its own measurement uncertainty, leading to variations in results between devices. Despite widespread use, comparative data on diagnostic performance, particularly for HIV, HBsAg, and HCV testing, remain limited. Laboratory professionals often struggle to define acceptable variation limits, impacting diagnostic accuracy, efficiency, cost-effectiveness, and patient outcomes [2].

Laboratory professionals routinely encounter challenges in determining acceptable limits of variation when results are generated from different analyzers. Such variability has implications not only for diagnostic accuracy but also for laboratory workflow efficiency, cost-effectiveness, and ultimately patient management. Establishing evidence-based acceptability thresholds is therefore essential to ensure consistency and reliability across instruments used in routine clinical diagnostics. A systematic, head-to-head evaluation of platforms is critical for identifying discrepancies and defining practical limits of agreement [3].

This study addresses this need by directly comparing the analytical performance of two CLIA-based analyzers manufactured by the same company in detecting HIV, HBsAg, and HCV markers. By evaluating paired patient samples on both systems and applying robust statistical methods, including Pearson correlation, Bland-Altman analysis, and bootstrapping, this research aims to generate objective, data-driven guidance for laboratory practice. Understanding the degree of concordance or divergence between these platforms can support informed decisions regarding their use in screening versus confirmatory workflows. Through this comparative approach, the study seeks to enhance the evidence base on CLIA instrument performance and contribute to improved quality assurance in clinical diagnostics.

## Materials and methods

Study design

This was a prospective study conducted at a single-center diagnostic laboratory, designed as a cross-sectional study, conducted in a diagnostic laboratory attached to a medical college in Bengaluru, India, analyzing two CLIA-based immunoassay instruments for HIV, HBsAg, and HCV detection. The laboratory policy tests one patient's serum sample daily on both instruments; data from January 2019 to December 2024 were analyzed. This policy of using one sample per day for inter-instrument comparison exceeds the recommendations by accreditation bodies (one or two samples once in three months). All investigations were performed in compliance with ethical guidelines and received approval from the Institutional Ethics Committee (Reg No. ECR/215/Inst/KA/2013/RR-22) through the protocol number (MSRMC/EC/AP-16/11-2018). Samples came from routine viral marker screening for pre-operative investigations. Hemolyzed, lipemic, or insufficient samples were excluded. Only complete, valid results from both analyzers were considered for analysis.

Testing platforms

The analyzers used in this study were fully automated chemiluminescent immunoassay (CLIA) systems designed for the qualitative detection of infectious disease markers. To evaluate the correlation and agreement between two eCLIA platforms (Vitros ECi and Vitros 3600; QuidelOrtho Corp., San Diego, CA) in detecting HIV, HBsAg, and HCV, and to establish data-driven acceptability limits for inter-instrument variability. These platforms employ proprietary reagents and operate on a sandwich immunoassay principle utilizing enhanced chemiluminescence and microwell technology. All samples were tested simultaneously on both analyzers using the same patient specimen to eliminate time-dependent degradation and ensure comparability.

Assay procedure

The non-reactive samples were randomly selected from routine pre‑operative viral marker screening requests, with one patient serum per day included as per laboratory policy, that met inclusion criteria (non‑hemolyzed, non‑lipemic, adequate volume) and were processed on both analyzers according to the respective manufacturer protocols. The samples were chosen by different technicians working in different shifts of the day to avoid selection bias. Results were reported as Signal-to-Cutoff (S/CO) ratios, indicating the reactivity level for each viral marker. All values were recorded and tabulated for subsequent statistical analysis. Internal quality control procedures and instrument calibrations were performed as recommended by the manufacturer to ensure the reliability and accuracy of results [4,5].

Statistical analysis

Descriptive statistics, including mean, standard deviation, and range, were calculated for all parameters. Pearson’s correlation coefficient (r) was used to assess the linear relationship between analyzer 1 (MT2) and analyzer 2 (MT3). Agreement between the two analyzers and potential systematic bias were evaluated using Bland-Altman plots. Normality of data distribution was assessed using the Jarque-Bera test. All analyses were performed using Microsoft Excel (Microsoft Corp., Redmond, WA) and SPSS v25 (IBM Corp., Armonk, NY) [6,7].

The bootstrapping analysis was used to calculate an operational benchmark for the upper limit of errors. Bootstrapping is a statistical procedure that resamples a single dataset to create many simulated samples. Each re-sampled dataset is used to calculate the statistic of interest, an out-of-limit variable, such as the mean or variance. Bias is considered to be out of the limits of the upper confidence interval of absolute differences between the measurements of the two methods. The confidence interval is based on the observed data. If a difference is more than the upper limit, then the value is defined as out of limit. Standard error was calculated as the standard deviation of the proportion of out-of-limits.

## Results

A total of 830 paired serum samples were analyzed on both eCLIA platforms (MT2 and MT3) for HIV, HBsAg, and HCV. After exclusion of incomplete and zero values, 828 HIV, 825 HBsAg, and 823 HCV paired results were included in the final analysis. Table 1 summarizes the final number of samples included, along with their mean and standard deviation values.

**Table 1:** Final number of samples included along with their mean and standard deviation values

Parameters	HIV		HBsAg		HCV	
	MT2	MT3	MT2	MT3	MT2	MT3
Count	828	828	825	825	823	823
Mean	0.140	0.156	0.179	0.173	0.083	0.079
SD	0.095	0.097	0.097	0.092	0.101	0.096
CV	67.5%	61.8%	54.5%	53.4%	121.5%	121.2%
Minimum	0.01	0.01	0.01	0.01	0.01	0.01
Maximum	0.85	0.9	0.91	0.88	1.03	1.16

Data variability and initial correlation assessment

**Figure 1:**
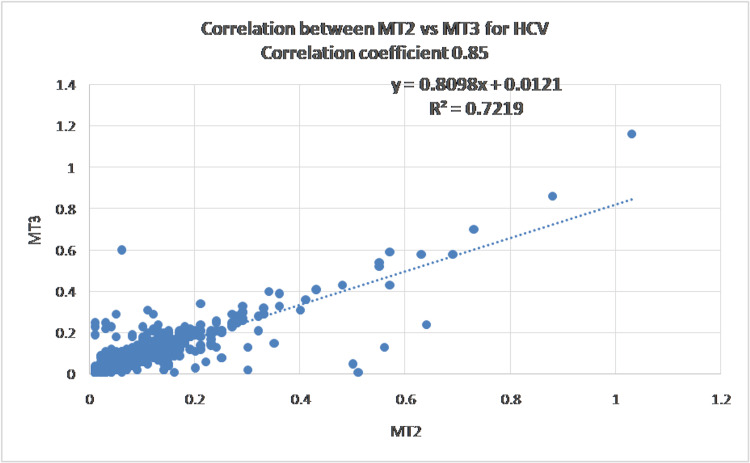
Scatter plot showing a strong linear association between Vitros ECi (MT2) and Vitros 3600 (MT3) for hepatitis C virus (HCV)

**Figure 2:**
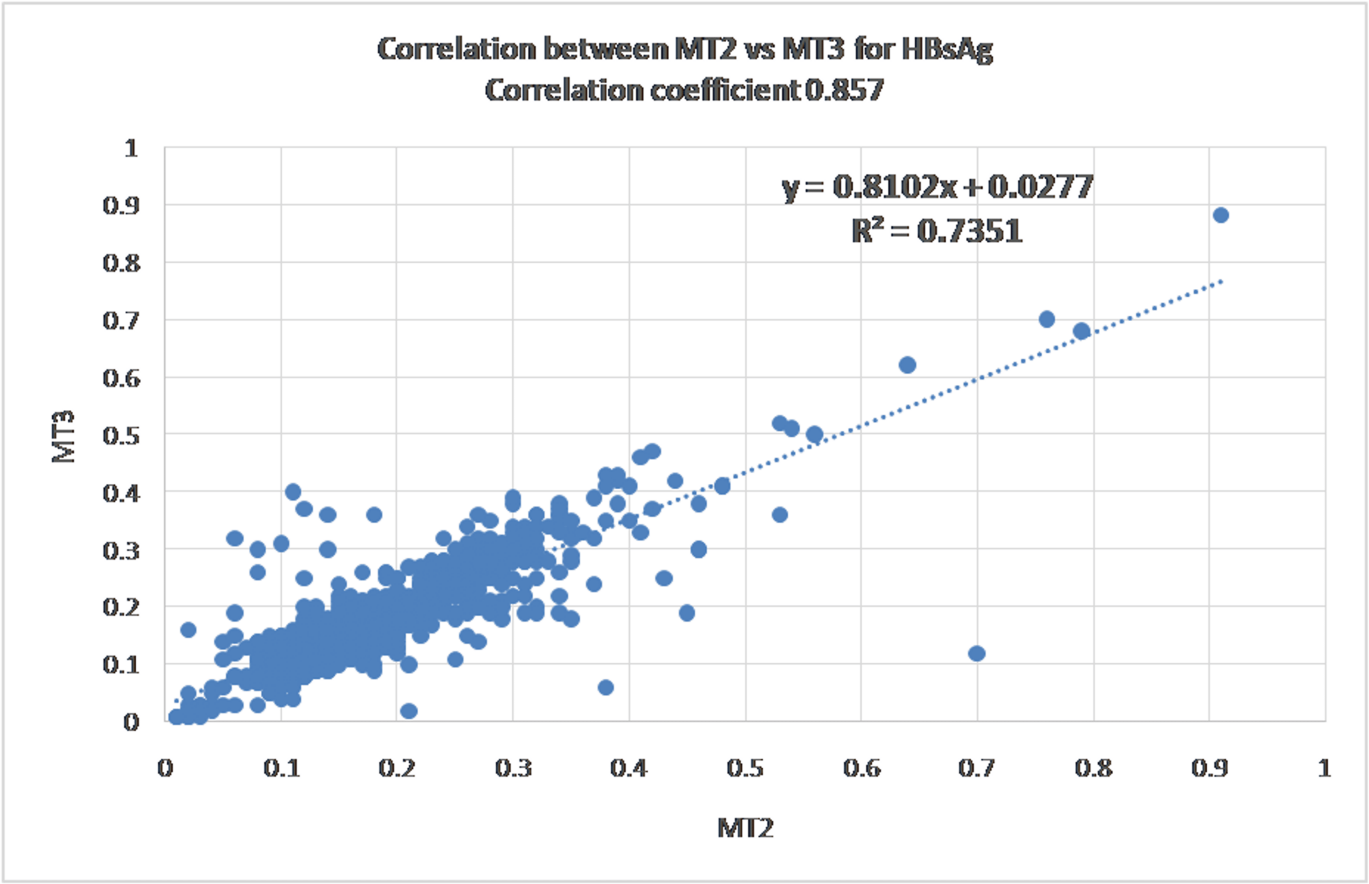
Hepatitis B surface antigen (HBsAg) results showing a moderate to strong correlation between the two analyzers

**Figure 3:**
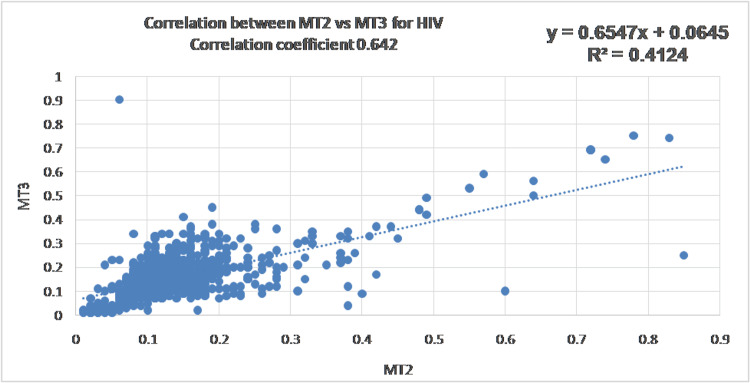
Scatter plot showing a positive correlation between Vitros ECi (MT2) and Vitros 3600 (MT3) for human immunodeficiency virus (HIV)

All three analytes demonstrated substantial variability, with coefficients of variation exceeding 50%, and HCV showing CV values greater than 100%. Pearson correlation analysis revealed strong correlations for HCV (r = 0.85, R² = 0.72) and HBsAg (r = 0.85, R² = 0.73), whereas HIV showed only moderate correlation (r = 0.64, R² = 0.41). Figures 1-3 show the scatter plot and Pearson's correlation for HCV, HBsAg, and HIV, respectively. 

Assessment of normality and implications for correlation analysis

**Figure 4:**
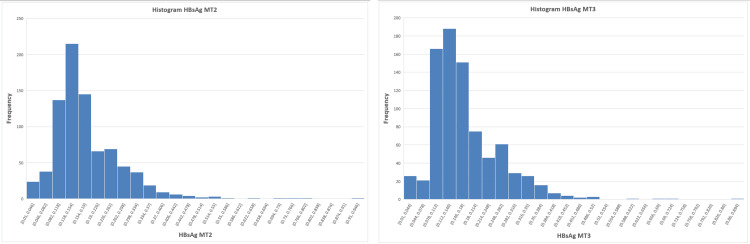
Histogram for hepatitis B surface antigen (HBsAg) showing deviations from normality in the underlying distribution

**Figure 5:**
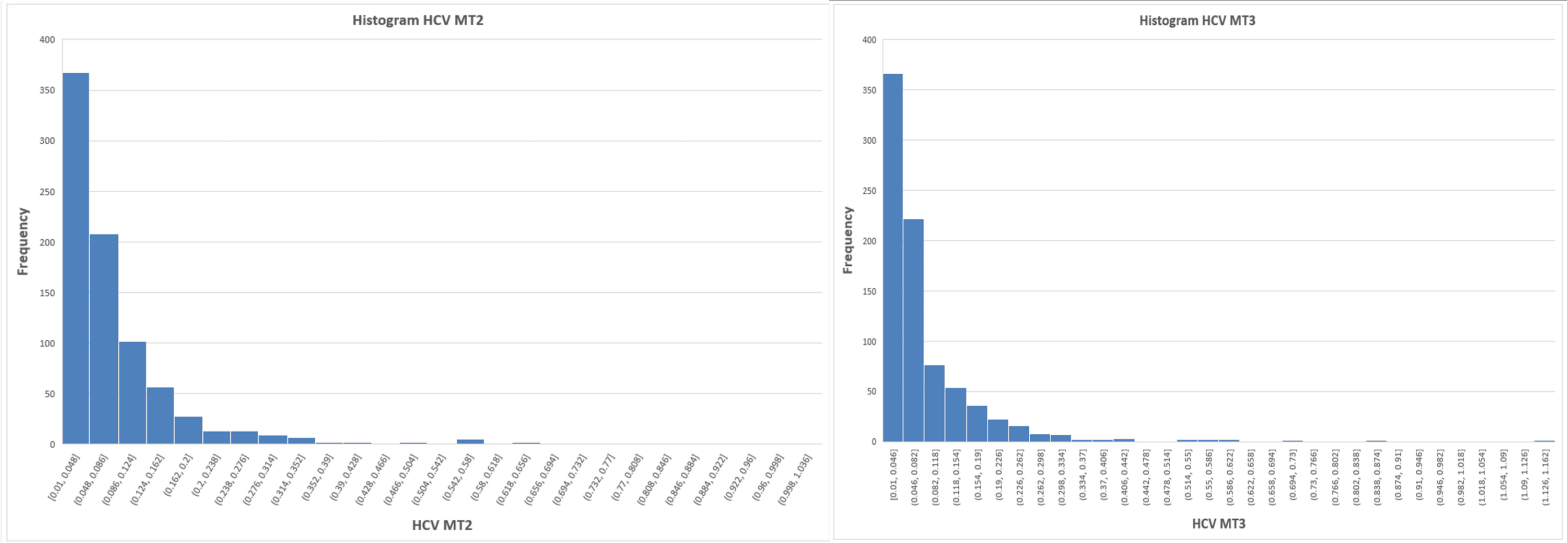
Histogram for hepatitis C virus (HCV) showing deviations from normality in the underlying distribution

**Figure 6:**
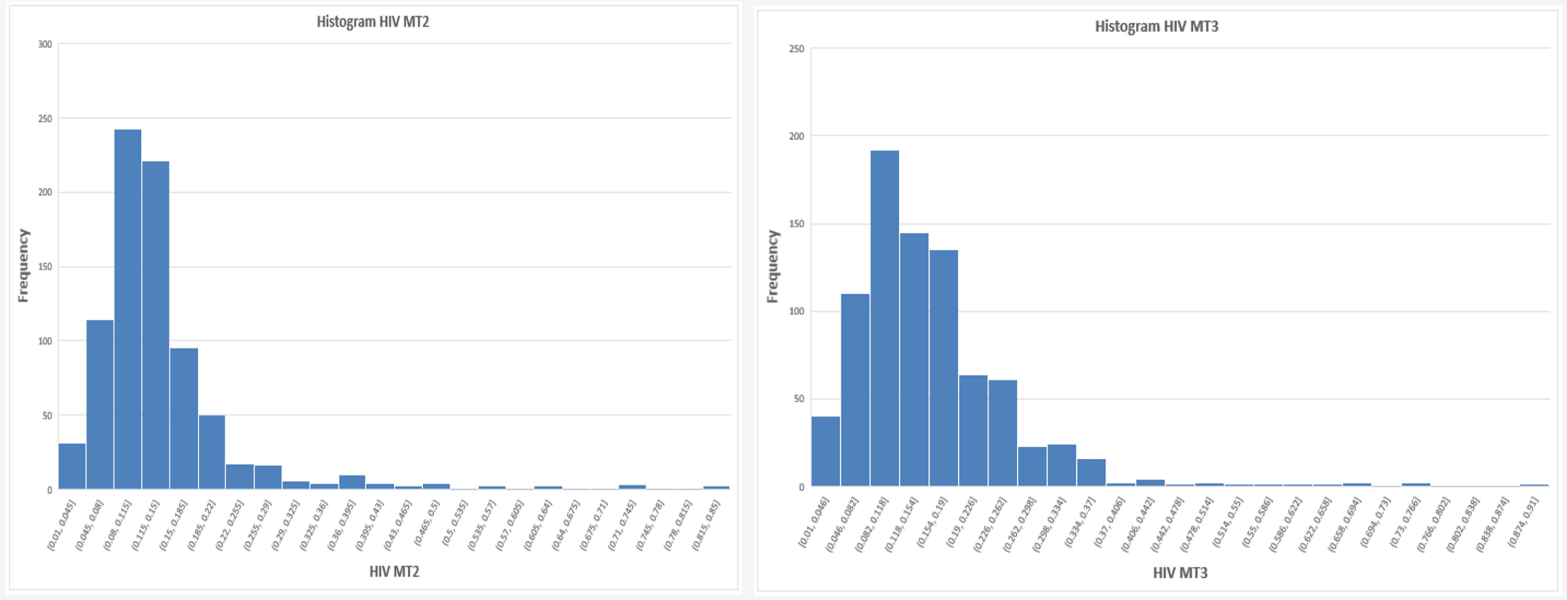
Histogram for human immunodeficiency virus (HIV) showing deviations from normality in the underlying distribution

To investigate the underlying distribution of results, histograms for each marker, as shown in Figures 4-6, were examined and indicated deviations from normality. This was further confirmed using the Jarque-Bera test, demonstrating significant deviation from normal distribution for all parameters (p < 0.0001), justifying the use of agreement-based statistical approaches.

Given that Pearson’s correlation assumes normally distributed variables, these findings indicate that correlation analysis alone may not be sufficient for evaluating agreement between analyzers (Table 2).

**Table 2:** Jarque-Bera analysis to check normality

Parameters	HIV		HBsAg		HCV	
	MT2	MT3	MT2	MT3	MT2	MT3
Count	828	828	825	825	823	823
Correlation	0.64		0.86		0.85	
Skew	3.57	2.38	2.08	1.84	3.98	4.35
Kurtosis	17.95	10.67	8.71	7.18	22.68	31.02
JB statistic	9471.95	2809.61	171.44	1066.16	15453.43	29507.90
p-value	<0.0001	<0.0001	<0.0001	<0.0001	<0.0001	<0.0001

Agreement analysis using Bland-Altman plots

**Figure 7:**
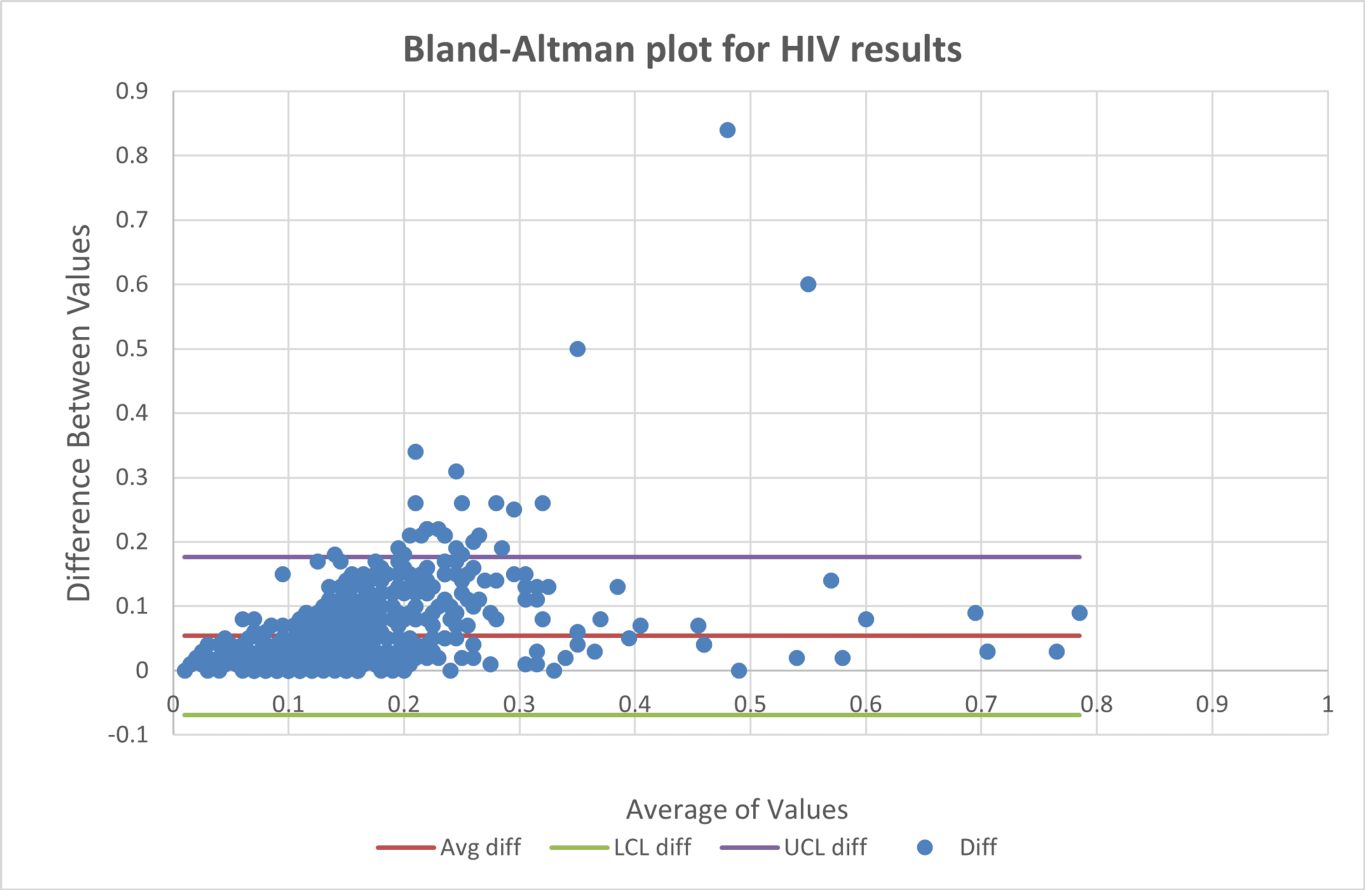
Bland-Altman plots for human immunodeficiency virus (HIV)

**Figure 8:**
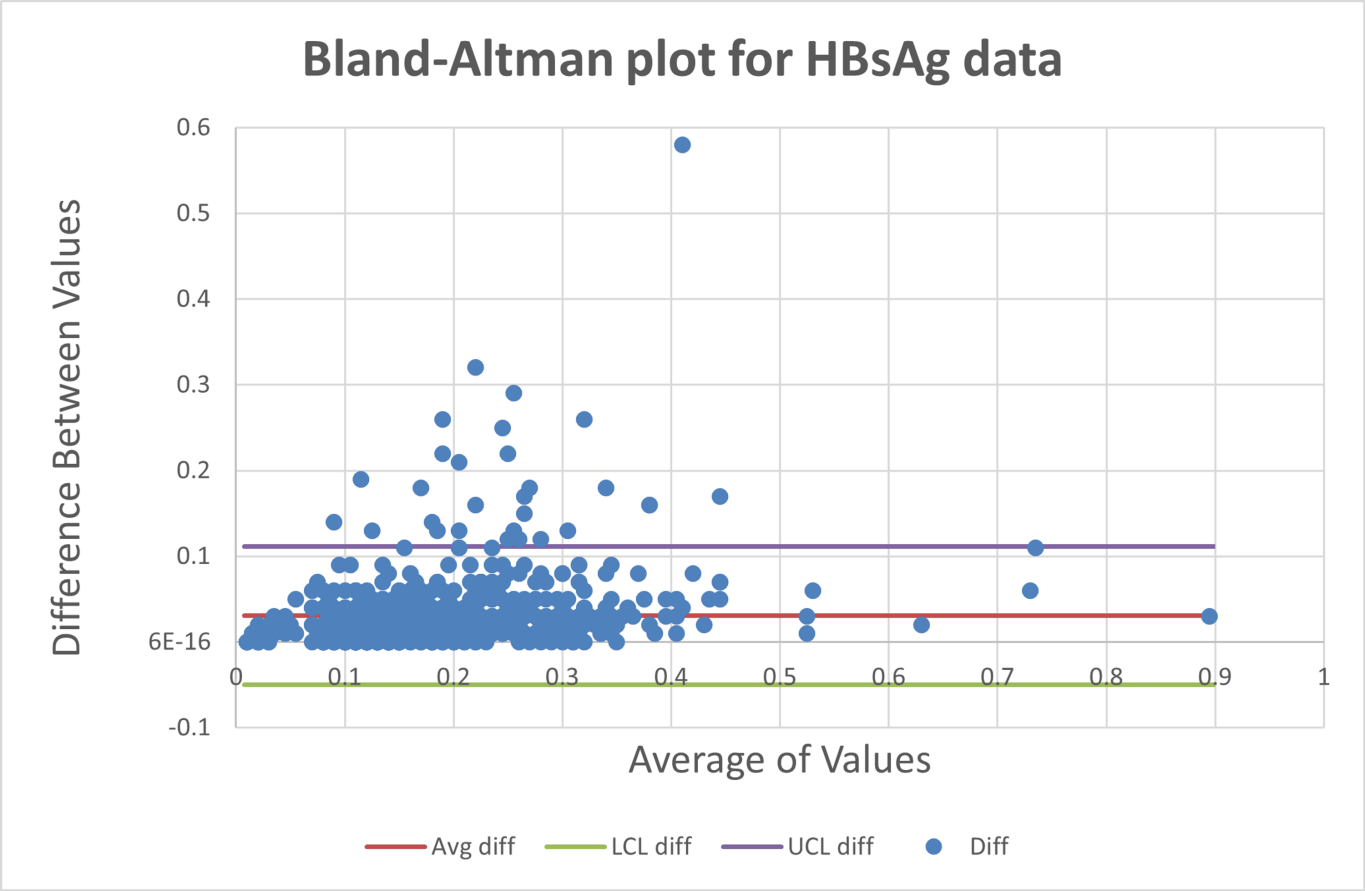
Bland-Altman plots for hepatitis B surface antigen (HBsAg)

**Figure 9:**
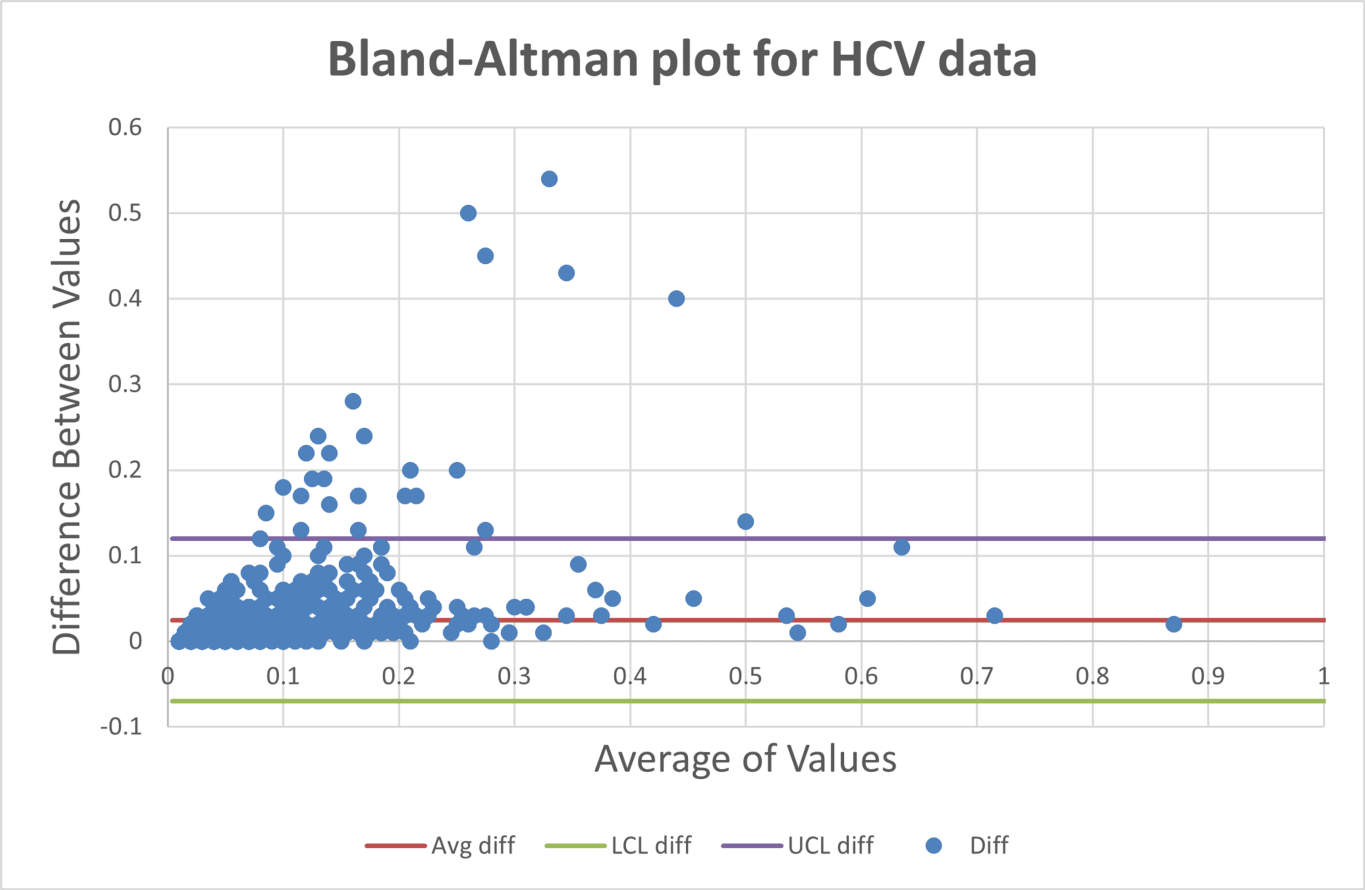
Bland-Altman plots for hepatitis C virus (HCV)

Bland-Altman analysis showed that approximately 95% of paired measurements for HIV (Figure 7), HBsAg (Figure 8), and HCV (Figure 9) fell within the limits of agreement, with out-of-limit values ranging from 3.36% to 3.65% as shown in Table 3.

**Table 3:** Bland-Altman limits of agreement for human immunodeficiency virus (HIV), hepatitis B surface antigen (HBsAg), and hepatitis C virus (HCV), with ~95% of paired differences within the limits of agreement (LoA).

	HIV	HBsAg	HCV
Number	828	825	823
Out of limits	27 (3.36%)	30 (3.64%)	30 (3.65%)
Agreements	801 (95.93%)	795 (95.32%)	793 (94.86%)

Bootstrapping analysis for estimating acceptability limits

Bootstrapping analysis (1000 iterations) demonstrated stable standard error estimates across resampled datasets, with upper error limits consistently ≤5% for all three analytes (Table 4), supporting the proposal of a 5% acceptability threshold for inter-instrument variability. Standard error is defined as the standard deviation of the proportion of out-of-limits. Out of limit value is defined as the value above the upper confidence interval of the absolute differences between the measurements of the two instruments.

**Table 4:** Bootstrap analysis @1000 iterations

Parameter	Upper limit of errors
HIV	4%
HBsAg	5%
HCV	4.6%

## Discussion

The present study evaluated inter-instrument variability between two enhanced chemiluminescence immunoassay (eCLIA) platforms for viral serology and aimed to define empirically derived acceptability limits in the absence of established industry standards. While chemiluminescent platforms are widely recognized for their high analytical sensitivity and automation, variability between instruments remains an important concern, particularly in laboratories operating multiple analyzers to meet accreditation and workflow requirements.

Correlation versus agreement in method comparison

Initial correlation analysis demonstrated moderate to strong linear relationships for HBsAg and HCV, consistent with previous reports evaluating CLIA-based platforms for viral serology [1,7]. However, HIV exhibited weaker correlation because the values did not follow normal distribution, highlighting that correlation coefficients are influenced by data range and distribution rather than true analytical concordance. This limitation has been well documented, as correlation measures association but not agreement between methods [5,6].

The markedly non-normal distribution observed across all analytes, confirmed by Jarque-Bera testing, further undermines the reliability of correlation-based interpretations. Viral serological data, particularly S/CO ratios, are inherently skewed due to the predominance of non-reactive samples clustered near the assay cutoff. Similar distributional challenges have been described in sero-surveillance and screening studies using chemiluminescent platforms [4].

Utility of Bland-Altman analysis in viral serology

Bland-Altman analysis provided a more clinically meaningful assessment of inter-instrument agreement by directly evaluating the magnitude and dispersion of paired differences. Despite differences in correlation strength, all three analytes demonstrated comparable agreement, with approximately 95% of values falling within the limits of agreement. This finding reinforces that high correlation does not necessarily translate to superior agreement, a misconception frequently encountered in laboratory method comparison studies [3].

Importantly, HIV, despite having the weakest correlation, showed a similar proportion of out-of-limit values compared to HBsAg and HCV. This suggests that analytical variability between instruments may be driven more by assay architecture, signal amplification dynamics, and cutoff proximity than by analyte-specific performance alone.

Defining acceptability in the absence of standards

While Bland-Altman plots effectively describe agreement, they do not define whether the observed limits are acceptable for clinical use. Acceptability must be determined based on predefined clinical, biological, or operational criteria (Bland and Altman, 1986) [5]. However, for qualitative or semi-quantitative viral serology assays reported as S/CO ratios, universally accepted allowable error limits are lacking.

Bootstrapping is a statistical technique that involves resampling a single dataset to create multiple simulated samples. This method is used to estimate the accuracy of statistics such as standard errors, confidence intervals, and hypothesis testing. The primary purpose of bootstrapping is to provide a more accurate and reliable way to make statistical inferences, especially when dealing with small sample sizes or unknown data distributions. Each resampled dataset is used to calculate the statistic of interest, out-of-limit variable, such as the mean or variance. By aggregating these statistics, an empirical distribution is formed, which helps in estimating confidence intervals and standard errors. The consistently low error rates (≤5%) across the three analytes and iterations indicate that observed inter-instrument differences are not random artifacts but represent a stable analytical characteristic of the platforms studied. Similar resampling-based approaches have been recommended for method comparison when traditional parametric assumptions are violated [3].

Implications for laboratory practice

From a practical laboratory perspective, a ≤5% inter-instrument variability threshold offers a realistic and evidence-based benchmark for assessing analyzer interchangeability. Such a threshold is unlikely to affect qualitative interpretation in routine screening but provides a safeguard for quality assurance, instrument validation, and accreditation audits. This is particularly relevant in high-throughput laboratories where patient samples may be processed interchangeably across multiple analyzers [8].

Furthermore, the findings underscore the importance of using agreement-based statistics rather than correlation alone when validating or troubleshooting diagnostic platforms. Laboratories relying solely on correlation metrics may underestimate clinically relevant discrepancies, especially for skewed serological data.

Limitations of the study

This study has a few limitations, being a single, site-specific investigation involving two analyzers from the same manufacturer, the findings may not be directly applicable to other CLIA platforms or laboratory environments. The inclusion of a single patient sample per day and the non-inclusion of reactive samples may have limited the assessment of inter-instrument variability, particularly at higher S/CO values. Additionally, discordant results were not subjected to clinical correlation, as the primary objective was to evaluate analytical agreement rather than diagnostic accuracy. Although bootstrapping was employed to derive an acceptable variability threshold, the proposed 5% limit is empirically based and needs to be confirmed in larger studies conducted across multiple centers and different analytical systems.

## Conclusions

In a contemporary clinical laboratory, it is common to evaluate the agreement between two methods, yet the appropriate statistical approach is not always straightforward. Correlation and regression analyses are often used; however, they assess the relationship between variables rather than the differences and, therefore, are not recommended for determining comparability between methods. The Bland-Altman plot provides a simple and effective means to evaluate bias based on mean differences and to estimate an agreement interval within which 95% of the differences between two methods fall. This method, however, only defines these intervals and does not indicate whether such limits are acceptable. Acceptable limits must be determined a priori, guided by clinical needs, biological considerations, or industry goals.

In this study, no predefined industry standards for acceptable limits were available. Therefore, bootstrapping analysis was applied to estimate an empirically supported threshold for acceptable error. Based on the bootstrapping results, the study supports a ≤5% acceptability threshold for inter‑instrument variability for Vitros ECi as a practical, laboratory‑specific benchmark. However, this proposed limit is derived from the present dataset and requires external validation before broader adoption. This threshold reflects a practical and statistically supported boundary for evaluating comparability between the two instruments.

## References

[1] Alonso R, López Roa P, Suárez M, Bouza E (2014). New automated chemiluminescence immunoassay for simultaneous but separate detection of human immunodeficiency virus antigens and antibodies. J Clin Microbiol.

[2] Sulaeman H, Grebe E, Dave H (2023). Evaluation of Ortho VITROS and Roche Elecsys S and NC immunoassays for SARS-CoV-2 serosurveillance applications. Microbiol Spectr.

[3] Silveira PS, Vieira JE, Siqueira JO (2024). Is the Bland-Altman plot method useful without inferences for accuracy, precision, and agreement?. Rev Saude Publica.

[4] Muñoz-Chimeno M, Valencia J, Rodriguez-Recio A (2023). HCV, HIV AND HBV rapid test diagnosis in non-clinical outreach settings can be as accurate as conventional laboratory tests. Sci Rep.

[5] Bland JM, Altman DG (1986). Statistical methods for assessing agreement between two methods of clinical measurement. Lancet.

[6] Giavarina D (2015). Understanding Bland Altman analysis. Biochem Med (Zagreb).

[7] Ismail N, Fish GE, Smith MB (2004). Laboratory evaluation of a fully automated chemiluminescence immunoassay for rapid detection of HBsAg, antibodies to HBsAg, and antibodies to hepatitis C virus. J Clin Microbiol.

[8] Vaid R, Sandeep T, Sanjeevi P (2021). Degree of agreement between Vitros ECi instruments in serological tests by Bland-Altman analysis. International Journal of Medical Sciences and Innovative Research.

